# Supplementary material for: HIVprotI: an integrated web based platform for prediction and design of HIV proteins inhibitors
Source: J Cheminform. 2018 Mar 9;10:12. doi: 10.1186/s13321-018-0266-y (PMC5845081; doi:10.1186/s13321-018-0266-y)
Supplement: Supplementary file 2 — Additional file 2. Source code of HIVProtI web server. [file 13321_2018_266_MOESM2_ESM.zip › HIVprotI_Source-code/hivprotI/weka-3-6-6/doc/weka/gui/package-frame.html]

weka.gui


weka.gui

Interfaces 
  
*CustomPanelSupplier*
  
*Logger*
  
*MainMenuExtension*
  
*TaskLogger*

Classes 
  
AttributeListPanel
  
AttributeSelectionPanel
  
AttributeSummaryPanel
  
AttributeVisualizationPanel
  
BrowserHelper
  
CheckBoxList
  
ComponentHelper
  
ConverterFileChooser
  
CostMatrixEditor
  
DatabaseConnectionDialog
  
ExtensionFileFilter
  
FileEditor
  
GenericArrayEditor
  
GenericObjectEditor
  
GenericPropertiesCreator
  
GUIChooser
  
GUIChooser.ChildFrameSDI
  
HierarchyPropertyParser
  
InstancesSummaryPanel
  
JListHelper
  
JTableHelper
  
ListSelectorDialog
  
Loader
  
LogPanel
  
LogWindow
  
LookAndFeel
  
Main
  
Main.BackgroundDesktopPane
  
Main.ChildFrameMDI
  
Main.ChildFrameSDI
  
MemoryUsagePanel
  
Messages
  
PropertyDialog
  
PropertyPanel
  
PropertySelectorDialog
  
PropertySheetPanel
  
ResultHistoryPanel
  
ResultHistoryPanel.RKeyAdapter
  
ResultHistoryPanel.RMouseAdapter
  
SaveBuffer
  
SelectedTagEditor
  
SetInstancesPanel
  
SimpleCLI
  
SimpleCLIPanel
  
SimpleCLIPanel.CommandlineCompletion
  
SimpleDateFormatEditor
  
SortedTableModel
  
SortedTableModel.SortContainer
  
SplashWindow
  
SysErrLog
  
ViewerDialog
  
WekaTaskMonitor
